# Institutional investors and cost of capital: The moderating effect of ownership structure

**DOI:** 10.1371/journal.pone.0249963

**Published:** 2021-04-08

**Authors:** Xiaoping Huo, Hongying Lin, Yanan Meng, Peter Woods

**Affiliations:** 1 Business School, Guilin University of Technology, Guilin, P.R. China; 2 Department of Business Strategy and Innovation, Griffith University, Brisbane, Australia; Shandong University of Science and Technology, CHINA

## Abstract

Guiding institutional investors to actively participate in corporate governance is a hot issue to improve the internal governance of China’s listed companies. This study seeks to provide a comprehensive understanding of the mechanism that underlies the governance effects of the heterogeneity of institutional investors on the cost of capital, and the influence of ownership structure on the relationship between them. Using an unbalanced panel data on A-share listed companies of Shanghai and Shenzhen in China’s capital market during the 2014–2019 period, this study reveals how institutional investors with longer holding period and higher shareholding ratio are negatively associated with the cost of capital in China’s capital market. Furthermore, this study successfully confirms the moderating effect of ownership structure in the relationship between institutional investors and the cost of capital. China’s state-owned enterprises are more likely to introduce improvements at the corporate governance level, and ownership concentration weakens the negative influence of institutional investors on the cost of capital. The research contributes to a deeper understanding of the impacts of institutional investor’s heterogeneity and ownership structure on the cost of capital in China. In the process, the study yields useful implications for the theory and practice of corporate governance.

## Introduction

Over the last two decades, under the rapid development of institutional investors’ strategic guidance, the number and scale of China’s institutional investors has significantly expanded. However, the precipitous growth of the size of institutional investors has exposed a multiplicity of problems, throwing into doubt the role of institutional investors in China’s capital market. As well, questions about the principles and policies of institutional investors’ development have been raised. With a new round of mixed ownership reform in China from 2013, state-owned enterprises are expected to implement the governance effects of mixed public-private ownership by introducing private capital [1]. As institutional investors are an important source of capital with immense financial strength and investment potential, the role played by institutional investors in the development of China’s state-owned enterprises deserves serious discussion.

At the end of 2016, a total of 16.3% of the market value of the Shanghai and Shenzhen stock markets in China was invested in public offerings, private funds, insurance, securities, trusts and so on [2]. In 2017, the China Securities Regulatory Commission (CSRC) stressed that China would standardize and expand channels for the entry of various funds, and develop long-term institutional investors. In 2019, a large scale fund system was established by the State-owned Assets Supervision and Administration Commission of China, to further entrench the reform of state-owned enterprises by attracting various funds, including institutional investors [3].

In 2018 the shareholding ratio of institutional investors was 31.5% in China, and 93.2% in the USA, in contrast to a shareholding ratio of individual investors of 40.5% in China, and 4.1% in the USA [4]. Gao et al. [5] researched some developed stock markets, such as United States, South Korea, Hong Kong and Taiwan, and they found that financial institutions in the United States continuously absorbed foreign assets and became the investor type with the highest proportion of assets in the American market, institutional investors occupy the main position in the proportion of shareholding market value in South Korea’s stock market and Hong Kong’s stock market, the stock markets of Taiwan and mainland China are typical markets dominated by individual investors, while the proportion of institutional investors is relatively low.

However, assets held by institutional investors in China are the fastest growing in the world [1]. Credit Suisse has predicted that it will rise from $1400 billion in 2014 to $13800 billion in 2030 [6, 7]. Compared with mature capital markets in the United States, institutional investors have become an important force in China’s current capital market. In recent decades, they own and hold voting rights across the majority of China’s capital markets’ equity capital, as well as being legal and professional entities that specialize in investment activities in the capital market. Compared to individual investors, they enjoy considerable advantages of capital, access to information and stronger professional capacities. Borochin and Yang [8] found that institutional investors have extremely robust information and data gathering capabilities, which can help improve market efficiency and asset valuation.

In recent years, the cost of capital has received greater attention from both practitioners and finance academics. From a microscopic point the cost of capital impacts on the enterprise value directly, and it exists throughout the overall financial policy making process. Moreover, from a macroscopic perspective, the cost of capital is the basic index for security system construction and capital market development [9]. Many firms devote a significant portion of their annual reports to analyze their cost of capital, and several issue annual cost of capital reports detailing the benefits derived from investment, financing and dividend policies. In 2010, the cost of capital started to be applied to evaluate senior executives of China’s state-owned enterprises by EVA (Economic Value Added), and in 2013, the proportion of EVA in evaluations was increased. Furthermore, an expanding body of academic research is analyzing the specific factors that influence the cost of capital [10]. However, relatively little is known about the influence on cost of capital by institutional investors in China’s capital market, since the size of institutional investors’ assets are growing fast, and the equity environment is undergoing distinct transformation under the recent reform of mixed ownership in China.

This study takes a different approach to shed light on the influence of institutional investors on the cost of capital. We consider the investment period and shareholding ratio of institutional investors, and test for a cross effect between institutional investors and the firms’ ownership structure. We test whether firms whose investors have longer period and higher shareholding ratio have lower cost of capital, more than firms whose investors have shorter period and lower shareholding ratio do. The results of previous studies agreed that there is a negative correlation between the quality of corporate governance and the cost of capital [11–14]. Therefore, to a certain degree, the lower cost of capital reflects the higher quality of corporate governance. As the cost of capital plays a core role in corporate governance [15], it is critically important to clarify the influence of institutional investors on the cost of capital, in order to define the governance role of institutional investors. Furthermore, all else being equal, firms with different ownership structures have different cost of capital. Extant research on governance has underlined the nature of ownership structure as foundational to the quality of relationships within firms [16]. It is necessary to consider the essential function of the equity environment when researching the influence of institutional investors on cost of capital. We therefore explore the extent to which ownership concentration and nature of equity may influence institutional investors’ effects on the cost of capital.

The remainder of the study proceeds as follows. Section 2 is a literature review as well as the corresponding research hypothesis. Section 3 describes the data and variable measurement, which introduce the data source and the variables used in this study. Section 4 is the empirical test of the influence of institutional investors on the cost of capital and the moderating effect of ownership structure. Section 5 is the robustness checks. Section 6 summarizes the conclusions and introduces policy recommendations.

## Literature review and hypothesis development

### Institutional investors and cost of capital

Although previous studies have focused on the investment preferences of institutional investors, the majority of these studies consider institutional investors as a homogeneous group with similar goals and investment strategies [17]. There are divergent views on whether institutional investors can effectively stabilize the market, and adequately supervise the controlling shareholders and managers–the absence of consensus possibly stems from the paucity of research that have taken as their focus on the influence of the heterogeneity of institutional investors [18]. Attig et al. and Garcia-Meca et al. pointed out the many variances among different types of institutional investors, such as asset size, information acquisition, risk preference, investment motivation, and value orientation [18, 19]. With this in mind, the general purpose of this study is to classify the institutional investors by type. Bushee divided institutional investors into three types, according to diligence, transience and quasi-index, and concluded that diligent institutional investors could reduce the short-sighted behaviors of managers [20], while transient institutional investors greatly influenced managers to reduce R&D, being more concerned with meeting short-term profit targets. Tang and Yuan divided listed companies into three types by high, medium and low institutional investors’ shareholding ratio [21]. They believed that institutional investors with higher share proportions acted as effective supervisors and those with lower share proportion acted as interest grabbing.

Institutional investors played an important role in the process of corporate governance, and institutional investors with higher share proportion could improve the quality of accounting information [22, 23], play an important role in shaping managerial behavior [24] and controlling the behaviors of earnings management [25, 26]. Institutional investors could alleviate the principal-agent problems between shareholders and managers to adjust the salary structure and the level of managers [27, 28] and reduce short-sighted behaviors of managers [29]. Filatotchev confirmed that institutional investors can impose different requirements in term of board independence [30]. Levitt argued that strong institutions improve the quality of accounting standards, therefore reducing the cost of capital [31]. The previous literature provides evidence on the role of institutional investors, indicating that institutional investors with higher proportion have better access and incentives to monitor managerial behavior, to secure a reduction in information asymmetry and to focus on the firm’s performance, and consequently decreasing the cost of capital.

**Hypothesis 1.** The cost of capital could be reduced more significantly by the institutional investors with a higher shareholding ratio than those with a lower shareholding ratio.

In this study, institutional investors were divided into long-term and short-term institutional investors, based on their investment value orientation. Long-term institutional investors who expect to get profits by dividends and value-added pay attention to investment values. They are more willing to participate in the firms’ management activities and to supervise managers to obtain greater benefits. However, short-term institutional investors are more concerned about the volatility of investment. They tend to exert influence on stock price, and force managers to pursue short-term benefits at the expense of long-term value and interests [32]. It would appear that institutional investors with different share proportion and value orientations are completely different in terms of their effects on firms’ cost of capital.

Research by Attig et al. showed that the presence of long-term institutional investors would lead to an improvement in monitoring and information quality, and a corresponding reduction in a firm’s cost of capital and information asymmetry problems [33]. Long-term and short-term institutional investors with different investment horizons and monitoring incentives have different governance roles [34]. Long-term institutional investors are more likely to persuade managers to enhance long-run value maximization and corporate innovation [35]. Moreover, long-term institutional investors are prone to monitoring costs and acting to control managers’ decisions in order to protect their interests in the firm [16]. Lakonishok et al. pointed out that an investor who bought and sold stock frequently would not obtain profits greater than the average income level, only long-term institutional investors with growth stocks would receive higher returns [36]. Coffee also believed that long-term institutional investors could obtain long-term gains due to improvements in corporate governance [37]. Therefore the longer holding period, the greater likelihood that institutional investors will play an effective supervisory role in corporate governance.

**Hypothesis 2.** The cost of capital could be reduced more significantly by the long-term institutional investors than the short-term ones.

### Ownership structure and cost of capital

Ownership structure is an important factor in determining the effectiveness of corporate governance. It not only affects the efficiency of corporate governance, but also relates to the operation and management in the entire capital market. Different ownership attributes of enterprises will lead to different resource endowments and governance structures, which will ultimately affect corporate governance efficiency and corporate performance [38]. Some scholars have proved that mixed ownership reform plays an important role in improving corporate governance level and corporate performance [39, 40]. The introduction of non-state-owned shareholders in state-owned enterprises can realize the combination of mechanism and resources, form a balanced and diversified ownership structure, and reduce agency conflicts [41]. Therefore, the consideration of the governance role of ownership structure is more fruitful for researching the effect on cost of capital by institutional investors especially under the circumstances created by mixed ownership reform in China today.

In line with prior research, we conclude that ownership concentration, one of several corporate governance mechanisms, significantly influences improvements of the supervisory role of shareholders toward managers [42, 43]. Based on principle-agent problems, Garmaise and Liu pointed out that the major proactive shareholders could supervise the managers, and force them to reduce over-investment, systematic risk and the cost of capital [39]. Bo and Wu studied the governance role of institutional investors from the perspective of earnings management, and they found that the governance role of institutional investors differed significantly according to the nature of equity involved [40].

Furthermore, we argue that institutional investors are generally composed of multiple agencies, and that there are significant differences in investment motivation, investment philosophy and investment strategies amongst the different types of institutional investors. Therefore, it is difficult for institutional investors to have a governance role in a listed company and to be in a controlling position. Gao and Zhang pointed out that it was difficult for institutional investors to play a strong supervisory role over the controlling shareholders under the background of a single listed company [41]. Kaldonski et al. found that long-term stable institutional investors can curb the behaviors of earnings management in single-class structure companies [44]. Therefore, the role of institutional investors in reducing the cost of capital will be affected by the ownership structure. Shleifer and Vishny confirmed that ownership dispersion will impede governance because owners with small proportion lack the incentives, and access to monitor individual managers [45].

Based on a review of the balance of power between large and small stakeholders, we conclude that the governance role of institutional investors is weakened in companies with higher ownership concentrations, for they have less voice in decision-making. However, institutional investors have a great influence on reducing the cost of capital and the improvement of corporate governance in companies with lower ownership concentration due to having a greater voice in decision-making. Based on the above analysis, we can infer that the impact of ownership structure and institutional investors on the cost of capital in the listed companies may have mutual promotion or substitution effect. In essence, the ownership concentration may possibly affect the relationship between institutional investors and cost of capital.

**Hypothesis 3.** The negative influence of institutional investors on the cost of capital could be weakened by ownership concentration.

We analyze the association between the nature of equity and the cost of capital of listed companies to assess the effectiveness of institutional investors’ monitoring of managers in China. State-owned enterprises are often larger in China, and managers are usually appointed by superiors, who manage the companies very efficiently, strive to raise and enhance the value of assets in order to achieve political advancement. Gourevich and Shinn suggested that as the ultimate controller of state-owned enterprises, the government often exerts political power to protect their own interests when entering into contracts with other shareholders [46]. Qian pointed out that state-owned enterprises gain greater government support in finance and policy decisions compared to non-state-owned enterprises [47]. Research by Li et al. showed that evidence from listed companies in China confirmed that the state-owned enterprises are more likely to introduce improvement at the corporate governance level [48]. Therefore, the cost of capital in state-owned enterprises is lower than in non-state-owned enterprises [49]. Previous studies suggest that institutional investors in state-owned enterprises are more able to fully assert regulatory governance to effectively monitor the behavior of state-owned shareholders that are not conducive to enterprise development.

**Hypothesis 4.** The cost of capital of state-owned enterprises decreases significantly with the increase of the shareholding ratio of institutional investors.

## Data and variable measurement

### Data and sample selection

We included in our study a sample of 10069 A-share listed companies on Shanghai and Shenzhen Stock Exchange in China during the period 2014–2019. We screened the non-balanced panel data according to the following principles: (1) Excluding samples with missing data; (2) Excluding samples with negative or greater ratio more than 1 of asset-liability ratio; (3) Excluding samples of financial and insurance industries; (4) Excluding samples of incomplete stock reform during the period of stock reform; (5) Excluding samples with extreme values; (6) Excluding samples with less than 1 year of listing time.

We selected a one-year lagged panel data of institutional investors to avoid the influence on the possibility that they may choose firms with lower cost of capital when investing, an eventuality that would be helpful in enabling us to uncover their effects on corporate governance [50, 51]. Moreover, to avoid the problems of endogenous and simultaneous effects, we also selected one-year lagged data of control variables. All data come from the China Stock Market & Accounting Research (CSMAR) Database.

In Table 1, we provide our definitions of the independent and dependent variables used in the analysis. In the following, we discuss how we measure cost of capital and briefly explain how to set up each variable in determining institutional investors’ heterogeneity.

**Table 1:** Variable definitions.

Variable	Definitions
*R*	Estimated value of the cost of capital by the OJ model.
*Insti*	The proportion of institutional investors shareholding.
*Insti_high*	The high-low dummy variables of institutional investors’ shareholding ratio: take the ratio’s median as a reference point. If the ratio is greater than or equal to the median, the value is 1. Otherwise, the value is 0.
*Insti_churn*	The long-short dummy variables of institutional investors’ shareholding period: the average annual turnover rate of listed companies with institutional shareholding is converted, choose its median as a reference point. If the value is greater than or equal to the median, the value is 0 which represents short-term institutional investors. Otherwise, the value is 1 which represents long-term institutional investors.
*Equil*	Ownership concentration: the shareholding ratio of the second to the tenth largest shareholders.
*State*	If the company is state-owned, the value is 1. Otherwise, the value is 0.
*Size*	Firm size: the natural logarithm of total assets.
*Lev*	Asset-liability ratio: the ratio of total liabilities to total assets.
*B /m*	Book to market: total assets/market value at the end of the period.
*Turn*	Total asset turnover: operating income / average total assets.
*Grow*	Operating sales growth: (operating income this year—operating income last year) / operating income last year.
*First*	Shareholding ratio of the largest shareholder
*Ind*	Independent directors’ ratio: the number of independent directors/ the number of the board of directors
*Dual*	CEO duality of chair and general manager: if the chair is general manager, the value is 1. Otherwise, the value is 0.
*Top*	Executive shareholding ratio: natural logarithm of executives’ shares
*Sal*	Executive compensation: natural logarithm of the top three executives’ total remuneration
*Year*	Dummy variable
*Industry*	Dummy variable

### Variable measurement

#### (1) Cost of capital

Cost of capital is the minimum rate of return required by investors to transfer the right to use capital. We construct the cost of capital variable using the implied cost of capital approach [52]. Existing estimating models of the cost of capital can be divided into two categories: prior estimation model with analyst forecast earnings’ data, and later estimation model with realized earnings replacing expected earnings [53], The implied cost of capital sets the estimation of cost of capital on the basis of forecast data rather than on the basis of historical data. Technically, it is to interpret the expected return level of investors implied in market prices, which is more in line with the “expected” characteristics of cost of capital–shareholders’ expectations of future investment returns. Mao et al. [54] points out that the implied cost of capital model can effectively reduce estimation errors and fully reflect the influence of various risk factors. Gode and Mohanram believed that the OJ model from the perspective of prior expected rates of return can reflect the level of market risk premium more significantly, and had less restrictive conditions when calculating the cost of capital [55]. The OJ model has the following three improvements: firstly, the data can be directly taken from the analyst’s forecast data; secondly, there is no need to forecast dividend payments; thirdly, there is no assumption of a clean surplus based on a per share price. The OJ model was proposed by Ohlson and Juettner [52], and it associates with the stock price, expected earnings and the growth of expected earnings. China’s stock market is relatively young, and stock prices are subject to fluctuation as it is affected by government intervention and major events that befall enterprises. In this study, we draw on the OJ model to calculate the cost of capital to make it more accurate. The standard evaluation model can be characterized by the following equations [56]:

$$R=A+\sqrt{A^{2}+{eps}_{1}(g_{2}-g_{p})/P_{0}}$$ (1)

A=12(gp+dps1/P0),g2=(eps2−eps1)/eps1 (2)

*R*: the cost of capital; *P*_0_: the opening price of a year; *dps*_1_: the dividend per share; *eps*_1_:the earnings per share; *eps*_2_: the earnings per share for the next year.

In the Eqs (1) and (2), *g*_*p*_ is the perpetual growth rate of the capitalized abnormal earnings growth, we determine it to be equal to the country-specific risk-free interest rate minus the country-specific long-term inflation rate or set equal to zero if negative [57]. According to Shen, risk-free interest rates takes 10-year bond yields and long-term inflation rate is 3%, *g*_*p*_ is replaced by 5% [58]. In the actual estimation process, if *eps*_1_>*eps*_2_, we set the short-term earnings growth (*eps*_2_−*eps*_1_) to zero. When the value inside the root is negative, we determine that *R* = *A* [59]. When selecting our sample, we excluded data from companies without opening prices and the earnings per share.

#### (2) Institutional investor

We construct our measure of institutional investors using the data obtained from the CSMAR Database in China. The shareholding period and ratio of institutional investors are expected to be crucial in determining the heterogeneity of investors. Short-term institutional investors are expected to trade their shares frequently, while long-term investors are expected to keep their portfolios unchanged over a considerable period of time. In this study, to test our hypothesis, the samples are divided into long-term and short-term subsamples by calculating turnover-rate, and then by measuring the frequency of which they rotate their portfolio stocks. Furthermore, the samples are also divided into higher shareholding ratio and lower shareholding ratio subsamples by measuring institutional investors’ shareholding ratio’s median value. We set up the long-short dummy variable of shareholding period named as *Insti*_*churn* and the high-low dummy variable of shareholding ratio named as *Insti*_*high* to measure.

#### (3) Contingency variables

We construct ownership structure variable using ownership concentration (*Equil*) and nature of equity (*State*)to identify the effect of ownership structure on the relationship between institutional investors and cost of capital.

Ownership concentration could be replaced by the shareholding ratio of the largest shareholder or the shareholding ratio of the second to the tenth largest shareholders according to some literatures [60, 61]. Since the largest shareholders of listed companies in China are mostly parent companies or ordinary legal persons, and there are fewer companies controlled by institutional investors [62], we adopt the shareholding ratio of the second to the tenth shareholders as the alternative index of ownership concentration while take the shareholding ratio of the largest shareholder as a control variable.

In the definition of nature of equity, the actual controller’s nature is taken from the data published in the “annual report”. The nature of equity of listed companies is classified according to the use guide of China’s listed companies’ shareholder research database (2013 edition), the name of the actual controller and the equity nature of the largest shareholder.

#### (4) Control variables

In our analyses, we control of other determinants that have been proven to effectively identify the effect of institutional investors on a firm’s cost of capital [10, 15, 63, 64]. These determinants are divided into two categories: financial variables and governance variables. Firm size (*Size*), measured as *the natural logarithm of assets*, is controlled in the regression analysis because large firms enjoy greater financial flexibility and lower uncertainty. Leverage is defined as *the ratio of total liabilities to total assets*, and is referred to as *Lev*. We also control of *the market-to-book equity ratio*, measured as *total assets/market value at the end of the period* and denoted *B /m*. Di Giuli et al. showed that market-to-book ratio can be used to measure financial distress, which affect a firm’s risk environment [65]. In addition, we include *Total Asset Turnover* and *Operating Sales Growth* in the regression, defined as *operating income / average total assets* and (*operating income this year—operating income last year) / operating income last year* individually. They are referred to as *Turn* and *Grow*. Moreover, corporate governance variables are included as control variables. *Shareholding ratio of the largest shareholder* is referred to as *First*. *Independent directors’ ratio*, measured as *the number of independent directors/ the number of the board of directors* and referred to as *Ind*. We use as the control variable *CEO duality of chair and general manager*, which we construct the value is 1 if the chair is general manager, otherwise, the value is 0. This variable is denoted *Dual*. In addition, executive compensation governance mechanism, which we define as *executive shareholding ratio* constructed *Top*, measured as *natural logarithm of executives’ shares* and as *executive compensation* constructed *Sal*, measured *natural logarithm of the top three executives’ total remuneration*. Variable definitions are shown in Table 1.

## Results and discussion

### Summary statistics

We report selected descriptive statistics for our variables in Table 2. Table 2 presents the summary statistics of all variables based on heterogeneous institutional investors groups. The samples are divided into four subsamples: higher shareholding ratio and lower shareholding ratio, longer shareholding period and shorter shareholding period. We compare whether two corresponding subsamples have significant differences in each of the main variables with T test methods and the non-parametric test method of median difference proposed by Mann Whitney.

**Table 2:** Analysis results of the main variables in different groups.

Panel A: Shareholding ratio of institutional investors							
Item	Samples with higher shareholding ratio(N = 5034)			Samples with lower shareholding ratio(N = 5035)			T-Test Value/Test Value
	Mean	median	SD	Mean	median	SD	
*R*	0.0697	0.0469	0.0582	0.0820	0.0466	0.0858	8.027***/1.532***
*Insti*	0.3008	0.2165	0.2302	0.2970	0.2231	0.2583	-1.760***/-6.463***
*Size*	22.2530	22.0949	1.2334	22.1492	21.9983	1.2759	-4.064***/-4.636***
*B /m*	0.4486	0.4065	0.2394	0.4849	0.4512	0.2539	7.216******/6.784***
*Turn*	0.6496	0.5509	0.4327	0.6283	0.5288	0.4275	-2.446***/-3.179***
*Lev*	0.4263	0.4154	0.2032	0.4187	0.4086	0.2076	-1.824***/-2.056***
*Grow*	0.2247	0.1278	0.4993	0.2006	0.0898	0.5225	-2.317***/-6.259***
Panel B: Shareholding period of institutional investors							
Item	Samples with longer holding period (N = 4962)			Samples with shorter holding period (N = 5107)			T-Test Value/Test Value
	Mean	Median	SD	Mean	Median	SD	
*R*	0.0710	0.0469	0.0634	0.0820	0.0466	0.0819	5.747***/2.268***
*Insti*	0.2323	0.1900	0.2302	0.1495	0.2522	0.2798	20.008***/9.892***
*Size*	22.7768	21.6814	1.0547	22.4119	22.2318	1.3038	24.808***/23.879***
*B /m*	0.4110	0.3759	0.2257	0.5017	0.4645	0.2547	17.684***/16.864***
*Turn*	0.6153	0.5185	0.4130	0.6484	0.5484	0.4380	3.677***/3.547***
*Lev*	0.3922	0.3774	0.2007	0.4187	0.4323	0.2068	10.594***/10.434***
*Grow*	0.2225	0.1208	0.5100	0.2039	0.1015	0.5150	-1.735***/-3.641***

***p<0.01, **p<0.05, *p<0.1.

Panel A shows that the mean value of the cost of capital of the sample of the listed companies with higher shareholding ratio (6.97%) is 1.23% less than one with lower shareholding ratio (8.20%). The cost of capital is negatively correlated with shareholding ratio of institutional investors. Panel B shows the mean value of the cost of capital of the samples of the listed companies with longer shareholding period (7.10%) is 1.10% less than one with shorter shareholding period (8.20%). The cost of capital is negatively correlated with shareholding period of institutional investors. Therefore, the higher shareholding ratio and the longer shareholding period of institutional investors, the more favorable the condition for the reduction of the level of the cost of capital of the listed companies.

### Regression analysis

#### (1) Baseline regressions

This study firstly explored the relationship between institutional investors and the cost of capital with the regression Model (1). The results show that the higher the shareholding ratio of institutional investors, the lower the cost of capital in column (1) and column (2) in Table 3.

$$R_{i,t}=a_{0}+\beta_{1}{Insti}_{i,t-1}+\beta_{2}{Size}_{i,t-1}+\beta_{3}B/m_{i.t-1}+\beta_{4}{Turn}_{i,t-1}+\beta_{5}{Lev}_{i,t-1}+\beta_{6}{Grow}_{i,t-1}+u_{i,t-1}+\varepsilon_{i,t-1}$$ Model (1)

**Table 3:** The effect of institutional investor on the cost of capital.

Variables	Coefficient					
	(1)	(2)	(3)	(4)	(5)	(6)
*C*	-2.9141 ***(-236.40)	-4.4332***(-24.79)	0.0820***(83.71)	-0.1050***(-3.38)	0.0802***(85.59)	-0.0988***(-5.60)
Insti	-0.0442***(-4.43)	-0.0259**(-2.35)				
Insti_high			-0.0124***(-8.03)	-0.0032***(-1.92)		
Insti_churn					-0.0091***(-5.75)	-0.0029*(-1.66)
*Size*		0.1017***(6.58)		0.0081***(9.14)		0.0076***(8.79)
*Lev*		0.0047 (0.37)		0.0054 (1.19)		0.0001 (0.02)
*Grow*		-0.0153 (-1.57)		-0.0002 (-1.31)		-0.0002***(-1.28)
*B/m*		0.0295* (1.83)		0.0120**(2.52)		-0.0132***(-3.07)
*Turn*		0.0302***(2.93)		0.0044***(3.07)		0.0045***(3.22)
*R2*	0.0019	0.0600	0.0063	0.0818	0.0033	0.0298
*F*	19.61***	24.58***	64.43***	34.39***	33.02***	51.54***
*N*	10069	10069	10069	10069	10069	10069

***p<0.01

**p<0.05

*p<0.1.

Next, to test Hypothesis 1, which predicts that firms with higher shareholding ratio of institutional investors have lower cost of capital, we estimate the following regression Model (2). To test Hypothesis 2, which predicts that firms with longer shareholding period of institutional investors have lower cost of capital, we estimate the following regression Model (3):

$$R_{i,t}=a_{0}+\beta_{1}{Insti\_high}_{i,t-1}+\beta_{2}{Size}_{i,t-1}+\beta_{3}B/m_{i.t-1}+\beta_{4}{Turn}_{i,t-1}+\beta_{5}{Lev}_{i,t-1}+\beta_{6}{Grow}_{i,t-1}+u_{i,t-1}+\varepsilon_{i,t-1}$$ Model (2)

$$R_{i,t}=a_{0}+Inst{i\_churn}_{i,t-1}+\beta_{2}{Size}_{i,t-1}+\beta_{3}B/m_{i,t-1}+\beta_{4}{Turn}_{i,t-1}+\beta_{5}{Lev}_{i,t-1}+\beta_{6}{Grow}_{i,t-1}+u_{i,t-1}+\varepsilon_{i,t-1}$$ Model (3)

Where, for firm *i* and year *t*, R represents the cost of capital which is dependent variable, and independent variables are the high-low dummy variables of institutional investor shareholding ratio (*Insti*_*high*) and the long-short dummy variables of institutional investors shareholding period (*Insti*_*churn*). The controlled variables include *Size*, *B*/*m*, *Turn*, *Lev* and *Grow*. *u* represents the year and industry dummy variables. We estimate the regression Model (2) and Model (3) with the fixed effect model method. The concrete results are shown in Table 3.

In Table 3, column (3) and column (4) show the effects on the cost of capital by the high-low dummy variables of the shareholding ratio of institutional investors. The column (5) and column (6) show the influence on the cost of capital by the long-short dummy variables of holding period of institutional investors. Table 3 shows that both the high-low dummy variables of the shareholding ratio of institutional investors and the long-short dummy variables of holding period of institutional investors have a significant negative correlation with the cost of capital. So institutional investors with higher shareholding ratio and longer holding period can play an active role in corporate governance and reduce the cost of capital effectively. Which is consistent with the research conclusion of Attig et al. [18]. Therefore, Hypothesis 1 and 2 of this study are confirmed.

#### (2) Moderating impacts

To test Hypothesis 3, which predicts that ownership concentration has a negative influence on the relationship between institutional investors and the cost of capital, we estimate the Model (4). To test Hypothesis 4, which predicts the cost of capital of state-owned enterprises is decreased more significantly than that of non-state-owned enterprises, we estimate the Model (5).

$$R_{i,t}=a_{0}+\beta_{1}{Insti}_{i,t-1}+\beta_{2}{Equil}_{i,t-1}+\beta_{3}{Insti}_{i,t-1}{Equil}_{i,t-1}+\beta_{4}{Size}_{i,t-1}+\beta_{5}B/m_{i,t-1}+\beta_{6}{Turn}_{i,t-1}+\beta_{7}{Lev}_{i,t-1}+\beta_{8}{Grow}_{i,t-1}+\beta_{9}{First}_{i,t-1}+\beta_{10}{Ind}_{i,t-1}+\beta_{11}{Dual}_{i,t-1}+\beta_{12}{Top}_{i,t-1}+\beta_{13}{Sal}_{i,t-1}+u_{i,t-1}+\varepsilon_{i,t-1}$$ Model (4)

$$R_{i,t}=a_{0}+\beta_{1}{Insti}_{i,t-1}+\beta_{2}{State}_{i,t-1}+\beta_{3}{Insti}_{i,t-1}{State}_{i,t-1}+\beta_{4}{Size}_{i,t-1}+\beta_{5}B/m_{i,t-1}+\beta_{6}{Turn}_{i,t-1}+\beta_{7}{Lev}_{i,t-1}+\beta_{8}{Grow}_{i,t-1}+\beta_{9}{First}_{i,t-1}+\beta_{10}{Ind}_{i,t-1}+\beta_{11}{Dual}_{i,t-1}+\beta_{12}{Top}_{i,t-1}+\beta_{13}{Sal}_{i,t-1}+u_{i,t-1}+\varepsilon_{i,t-1}$$ Model (5)

Where, for firm *i* and year *t*, dependent variable is the cost of capital (*R*), and independent variables are the interaction terms between the shareholding ratio of institutional investors and ownership concentration (Insti*Equil), and the interaction terms between institutional investors’ shareholding ratio and nature of equity (Insti*State). The controlled variables include *Size*, *B/m*, *Turn*, *Lev*, *Grow*, *First*, *Ind*, *Dual*, *Top* and *Sal*. *u* represents the year and industry dummy variables.

In order to investigate the interaction of ownership concentration and institutional investor on the cost of capital, we adopt Model (4) and Model (5) to test the interaction of ownership concentration (nature of equity) on the cost of capital with institutional investors based on the test methods of interaction effect proposed by Wen et al. [66]. There is a large multi-collinearity between product terms and low-order terms inevitably because product terms of ownership concentration and the shareholding ratio of institutional investors come from the values of two independent variables in the equation. Therefore, we utilize centralized treatment to reduce multi-collinearity from the polynomial or interactive effects model proposed by Hamilton which usually gets a more accurate coefficient estimate with smaller standard errors [67]. The empirical results are shown in Table 4, and column (1) and column (6) show the results of the full samples analysis. The analysis results on the interaction of ownership structure and institutional investors’ shareholding ratio are shown from the column (2) to the column (5) based on the classification of shareholding ratio of institutional investors and nature of equity.

**Table 4:** The effect on the cost of capital of ownership structure and institutional investors.

Variables	Full Sample	Full Sample				Full Sample
		Insti_high = 1	Insti_high = 0	State = 1	State = 0	
	(1)	(2)	(3)	(4)	(5)	(6)
C	-0.0783**(-2.42)	0.0394 (0.61)	-0.2022**(-2.03)	-0.1861***(-2.21)	-0.1348***(-4.20)	-0.0846***(-2.63)
Insti	-0.0116*(-1.79)	-0.282***(-3.24)	-0.0033 (-0.36)	-0.1000 (-0.98)	-0.0056 (-0.61)	-0.0079**(-2.37)
Insti* Equil	0.0459**(2.05)	0.0942 (3.02)	0.0261*** (0.86)	0.0902 (2.40)	0.0170* (0.56)	
Insti* State						-0.0005**(-2.34)
First	0.0002***(3.25)	0.0000 (0.31)	0.0003*** (3.02)	0.0002** (2.45)	0.0001 (1.64)	0.0001** (2.44)
Equil	-0.0001 (-1.18)	-0.0004***(-3.12)	-0.0000 (-0.23)	-0.0003 (-1.46)	-0.0001 (-0.95)	0.0001 (0.33)
State	-0.0030 (-1.55)	0.0005 (0.24)	-0.0059** (-2.11)			-0.0028 (-1.01)
Dual	-0.0029*(-1.66)	-0.0014 (-0.65)	-0.0044* (-1.67)	-0.0019 (-0.45)	-0.0039** (-2.02)	-0.0028* (-1.60)
Ind	0.0087 (1.15)	0.0118 (0.76)	0.0076 (0.82)	0.0078 (0.57)	0.0085 (0.92)	0.0087 (1.14)
Top	-0.0001 (-0.80)	-0.0002 (-1.38)	0.0000 (0.11)	0.0001 (0.49)	-0.0002 (-1.42)	-0.0001 (-1.01)
Sal	0.0030***(3.29)	0.0028** (2.42)	0.0028** (2.16)	0.0040** (2.56)	0.0026** (2.32)	0.0030***(3.33)
Size	0.0062***(6.25)	0.0018 (1.51)	0.0113*** (7.83)	0.0054***(3.51)	0.068*** (5.09)	0.0063***(6.43)
Lev	0.0077* (1.67)	0.0086 (1.48)	0.0060 (0.92)	0.0169** (2.17)	0.0025 (0.44)	0.0076* (1.66)
Grow	-0.0002 (-1.29)	-0.0002 (-1.28)	-0.0001 (-0.19)	0.0001 (0.22)	-0.0002 (-1.28)	-0.0001 (-1.23)
B/m	0.0166***(3.37)	0.0580***(8.90)	0.0072 (1.03)	0.0239***(3.17)	0.0054 (0.80)	0.0165***(3.33)
Turn	0.0036**(2.49)	0.0002 (0.15)	0.0060***(2.80)	0.0024 (0.92)	0.0045***(2.65)	0.0036** (2.47)
R^2^	0.0843	0.1050	0.0829	0.0903	0.0849	0.0839
F	27.16***	13.96***	15.38***	11.27***	17.57***	27.02***
N	10067	5034	5033	3780	6287	10067

***p<0.01

**p<0.05

*p<0.1.

Column (1) of Table 4 shows that the interaction variable Insti*Equil is significantly positively correlated with the cost of capital, while the share proportion of institutional investors has a significant negative correlation with the cost of capital on the empirical result of the full samples analysis. This suggests significant interaction between ownership concentration and the shareholding ratio of institutional investors on the cost of capital, and that ownership concentration weakens the negative influence on the cost of capital by the shareholding ratio of institutional investors. Therefore, Hypothesis 3 of this study is confirmed.

The results in column (2) and column (3) of Table 4 show that the interaction variable Insti*Equil is more significant in samples with the lower shareholding ratio of institutional investors, which means the interaction between ownership concentration and the shareholding ratio of institutional investors is mainly reflected in the samples with the lower shareholding ratio. Institutional investors with lower share proportion acquire fewer earnings, so they pay less attention to corporate governance and behaviors of managers. Compared to the listed companies with higher shareholding ratio, ownership concentration plays a more prominent governance role in the listed companies with lower shareholding ratio of institutional investors.

The results in the column (4) and column (5) of Table 4 show that the interaction term Insti*Equil is not significant in the samples which are divided into state-owned enterprises and non-state-owned enterprises. Wen et al. believed that the interaction can be considered significant when the independent variable coefficients (absolute value) increase while the interaction item is added [66]. Therefore, we analyze the change of independent variable coefficients when the interaction term Insti*Equil is added, and the results show that coefficients of independent variable *Insti* increase in the non-state-owned enterprises. It can therefore be considered that there is significant interaction between ownership concentration and the shareholding ratio of institutional investors. Ownership concentration weakens the effect of the shareholding ratio of institutional investors on the cost of capital in the non-state-owned enterprises. Compared with the state-owned enterprises, ownership concentration plays a more prominent governance role in the non-state-owned enterprises.

The results in column (6) of Table 4 show that there is a significant negative correlation between the interaction term Insti*State and the cost of capital. Marginal impact of the shareholding ratio of institutional investors on the cost of capital is (-0.0079–0.0005 *State*)*Insti under the same conditions, which indicates that the effects of institutional investors on decreasing the cost of capital are enhanced in the state-owned enterprises. From the statistical value, the negative effect of institutional investor on the cost of capital in the state-owned enterprises is greater than in the non-state-owned enterprises. So, Hypothesis 4 is confirmed.

The results above indicate that there are interactive effects on the cost of capital between ownership structure and institutional investor shareholdings. The cross term of the nature of equity and the shareholding ratio of institutional investors is significantly negative on the coefficient of the cost of capital, which means that institutional investor shareholdings have a larger negative influence on the cost of capital in the state-owned enterprises. The conclusion is consistent with the research of Dai [68]. The cross term of the ownership concentration and the shareholding ratio of institutional investors is significantly positive on the coefficient of the cost of capital. There is significant difference among the subsamples. But overall, ownership concentration weakens the negative influence of institutional investor shareholdings on the cost of capital, and the weakening effect is strengthened in the non-state-owned enterprises and some other enterprises with lower shareholding ratio of institutional investors.

### Robustness checks

In the empirical study of the study, we adopted the OJ model to calculate the cost of capital, which uses the analyst forecast earnings’ data. In the robustness checks, we use the Capital Asset Pricing Model (CAPM) to estimate the cost of capital, which uses historical data. This study uses the OJ model from the perspective of ex ante expected rate of return and the CAPM model from the perspective of ex post expected rate of return to estimate the cost of capital of listed companies in China, so that the research conclusion of the study could be more objective and robust. CAPM model is a very common method used by business community to estimate the cost of capital [69]. The following is the formula:

$$R=R_{f}+\beta (R_{m}-R_{f})$$ (3)

Where *R* is the firm’s cost of capital; *R*_*f*_ is risk-free rate of return, which is equal to one-year fixed deposit rate; *β* is Beta risk factor; *R*_*m*_−*R*_*f*_ is market risk premium.

We tested the relationship between the high-low dummy variables of the shareholding ratio of institutional investors (the long-short dummy variables of the shareholding period of institutional investors) and the cost of capital. The results in Table 5 are consistent with the above.

**Table 5:** The robustness test results.

Variables	Coefficients			
	(1)	(2)	(3)	(4)
C	0.0881***(22.67)	0.1763***(26.21)	0.0832***(21.27)	0.1726***(25.68)
Insti_high	-0.0051***(-11.86)	-0.0041***(-9.81)		
Insti_churn			-0.0023***(-6.49)	-0.0009**(-2.53)
Size		-0.0041***(-21.03)		-0.0043***(-21.57)
Lev		0.0072***(6.87)		0.0073***(6.93)
Grow		-0.0000 (-1.01)		-0.0000 (-1.12)
B/m		0.0032***(3.17)		0.0012***(3.81)
Turn		0.0012***(3.71)		0.0045***(3.22)
R^2^	0.0809	0.1283	0.0732	0.1216
F	46.74***	63.49***	41.93***	59.71***
N	11707	11707	11707	11707

**p<0.01

**p<0.05, *p<0.1.

We then tested the effect of interaction between institutional investors and ownership structure on the cost of capital. The samples are divided into the higher shareholding ratio of institutional investors and the lower one, and further divided into state-owned enterprises and non-state-owned enterprises based on the nature of equity. All variables are centrally processed and employed with the same methods of Tables 3 and 4. The results show that there is significant negative influence on the cost of capital by the shareholding ratio of institutional investors, which is more significant in the state-owned enterprises. Therefore, institutional investors can play a more positive and effective governance role in the state-owned enterprises. Similarly, there is significant interaction between institutional investors’ shareholding ratio and ownership concentration, and ownership concentration weakens the negative effect of institutional investor shareholdings on the cost of capital, which is mainly reflected in the non-state-owned enterprises and the ones with lower shareholding ratio of institutional investors. In summary, the results of the robustness test are almost consistent with the previous study (Tables 5 and 6).

**Table 6:** The robustness test results.

Variables	Full Sample	Full Sample				Full Sample
		Insti_high = 1	Insti_high = 0	State = 1	State = 0	
	(1)	(2)	(3)	(4)	(5)	(6)
C	-0.0199***(-3.68)	-0.0060 (-1.02)	-0.0132**(-2.27)	-0.0154***(-2.60)	-0.0265**(-2.48)	-0.0205***(-3.78)
Insti	-0.0238***(-6.02)	-0.0277***(-3.03)	0.0179 (0.95)	-0.0193*** (-2.96)	-0.0262***(-4.78)	-0.0067***(-6.55)
Insti* Equil	0.0216***(4.25)	0.0307 (2.37)	0.0341** (1.40)	0.0176 (2.27)	0.0272***(3.74)	
Insti* State						-0.0019*(-1.36)
R^2^	0.1395	0.1215	0.1606	0.1658	0.1452	0.1383
F	55.05***	24.03***	33.89***	25.77***	37.07***	54.51***
N	11707	5854	5853	4238	7434	11707

***p<0.01

**p<0.05

*p<0.1.

## Conclusions and policy recommendations

This study represents an empirical investigation of the effects of different types of institutional investors on the cost of capital, and the interaction of institutional investors and ownership structure on the cost of capital. The shareholding ratio of the institutional investors has negative effects on the cost of capital. Institutional investors with the higher shareholding ratio have greater negative effect on the cost of capital, and the longer holding period, the more favorable the conditions to decrease the level of the cost of capital. There is a significant interaction between institutional investor shareholdings and ownership concentration on the cost of capital. Ownership concentration weakens the negative effect of institutional investor shareholdings on the cost of capital, which is mainly reflected in the non-state-owned enterprises and others with lower shareholding ratio of institutional investors. Compared with the non-state-owned enterprises, the negative influence of institutional investors on the cost of capital is more significant in the state-owned enterprises. Institutional investors can play a more positive and effective governance role in the state-owned enterprises.

The study provides a reference for the Chinese government and professionals that will aid in forming policy as follows:

Firstly, the Chinese government should broaden the investment range of institutional investors and increase the share proportion of institutional investors. Although institutional shareholding in China has increased in recent years, in view of the overall situation, small retail investors account for the main actors in investment transactions in China’s A-share market. In this context, professional institutional investors become passive recipients of the market trend, and it is difficult for them to play a market stabilizer role. Therefore, accelerating the development of institutional investors and increasing the shareholding ratio of institutional shareholders are of great significant measures to entrench the reform of China’s state-owned enterprises.

Secondly, the innovation and development of fund management companies should be vigorously promoted. It should broaden industry access and encourage all kinds of qualified private capital and private funds to invest in public funds. Fund management firms should be encouraged to carry out the reform of mixed ownership and optimize the ownership structure. In addition, the Chinese government, policy makers and developmental agencies should formulate support plans for eligible institutions to independently develop public offering fund products to meet market demand, and encourage them to innovate in investment scope, marketing and strategy.

Thirdly, institutional investors should be encouraged to participate in the reform of state-owned enterprises to exert their positive regulatory and governance effects. Based on the analysis of this study, institutional investors have better governance effects on state-owned companies. Therefore, relevant policies in China should be taken as an opportunity to attract more non-state capital–including institutional investors–to participate in the development of the mixed ownership economy. Furthermore, the Chinese government should also guide institutional investors to establish a long-term investment philosophy concept, accelerate the construction of flexible financial market infrastructure, improve the long-term shareholding system of institutional investors, and promote institutional investors to properly establish the long-term value of the portfolio.

## Supporting information

S1 Dataset Empirical research data -OJ model for R. (XLSX)

S2 Dataset Robustness check data-CAPM model for R. (XLSX)

S3 Dataset Calculating cost of capital-OJ model+CAPM model. (XLSX)
